# Biosynthesis of Silver Nanoparticles Using Brown Marine Macroalga, *Sargassum Muticum* Aqueous Extract

**DOI:** 10.3390/ma6125942

**Published:** 2013-12-18

**Authors:** Susan Azizi, Farideh Namvar, Mahnaz Mahdavi, Mansor Bin Ahmad, Rosfarizan Mohamad

**Affiliations:** 1 Department of Chemistry, Faculty of Science, Universiti Putra Malaysia, 43400 UPM Serdang, Selangor, Malaysia; E-Mail: mansorahmad@upm.edu.my; 2 Institute of Tropical Forestry and Forest Products (INTROP), Universiti Putra Malaysia, 43400 UPM Serdang, Selangor, Malaysia; E-Mail: farideh.namvar@gmail.com; 3 Department of Medicine, Applied Biology Research Centre, Mashhad Branch, Islamic Azad University, Mashhad 917568, Iran; 4 Department of Chemistry, Faculty of Science, Shiraz Branch, Islamic Azad University, Shiraz 71993-3, Iran; 5 Department of Bioprocess Technology, Faculty of Biotechnology and Biomolecular Sciences, Universiti Putra Malaysia, 43400 UPM Serdang, Selangor, Malaysia; E-Mail: farizanmohd@gmail.com

**Keywords:** silver nanoparticles, *Sargassum muticum*, marine algae, biosynthesis

## Abstract

Biological synthesis of nanoparticles is a relatively new emerging field of nanotechnology which has economic and eco-friendly benefits over chemical and physical processes of synthesis. In the present work, for the first time, the brown marine algae *Sargassum muticum* (*S. muticum*) aqueous extract was used as a reducing agent for the synthesis of nanostructure silver particles (Ag-NPs). Structural, morphological and optical properties of the synthesized nanoparticles have been characterized systematically by using FTIR, XRD, TEM and UV–Vis spectroscopy. The formation of Ag-NPs was confirmed through the presence of an intense absorption peak at 420 nm using a UV–visible spectrophotometer. A TEM image showed that the particles are spherical in shape with size ranging from 5 to 15 nm. The nanoparticles were crystalline in nature. This was confirmed by the XRD pattern. From the FTIR results, it can be seen that the reduction has mostly been carried out by sulphated polysaccharides present in *S. muticum*.

## 1. Introduction

In the fields of nanoscience and nanotechnology, the largest activity has been focused on the synthesis of new nanoparticles with different sizes and new shapes, which have strong effects on their widely varying properties. Nanoparticles are attracting increasing attention due to their unusual and fascinating properties, which are strongly influenced by their size, morphology and structure [1].

Nanoparticles (NPs) are small in diameter, but large in surface area, and are important to many current exclusive medical and industrial applications such as biological engineering, catalysts, and electronic devices [2]. Currently, a large number of physical, chemical, biological, and hybrid methods are available to synthesize different types of nanoparticles [3]. Though physical and chemical methods are more popular for nanoparticle synthesis, the use of toxic compounds limits their applications. To overcome the problem of toxicity in synthesis, safe eco-friendly green methods have a major role for producing nanoparticles [4]. Several methods have been used for the green synthesis of NPs using various biological materials as reducing agents such as microorganisms, marine organisms, micro-fluids, and plant extracts [5,6,7,8,9]. Among the most important bioreductants are plant extracts, which are relatively easy to handle, readily available, low cost, and have been well explored for the green synthesis of other nanomaterials [10]. Moreover, the biologically active molecules involved in the green synthesis of NPs act as functionalizing ligands, making these NPs more suitable for biomedical applications [11]. Marine algae are well-known as functional food for their richness in lipids, minerals and certain vitamins, and also several bioactive substances like polysaccharides, proteins and polyphones, with potential medicinal uses against cancer, oxidative stress, inflammation, allergy, thrombosis, lipidemia, hypertensive and other degenerative diseases [12,13,14,15,16,17,18]. Thus, their phytochemicals include hydroxyl, carboxyl, and amino functional groups, which can serve both as effective metal-reducing agents and as capping agents to provide a robust coating on the metal nanoparticles in a single step [19].

Silver nanoparticles have been widely used during the past few years in various applications due to their well-known effectiveness in biomedical [20], electronic [21], catalysis [22] and optical applications [23]. In particular, the outstanding antimicrobial properties of Ag-NPs have led to the development of a wide variety of nanosilver products, including nanosilver-coated wound dressings, contraceptive devices, surgical instruments, and implants [24,25]. Apart from these antimicrobial activities, Ag-NPs are also known to possess antifungal, anti-inflammatory, antiviral, anti-angiogenesis, and antiplatelet properties [26,27]. Additionally, more recent developments have seen Ag-NPs used in room spray, wallpaper gloves, laundry detergent, and wall paint formulations as well as in the textile industry for clothing manufacturing [28,29].

The present study describes a single step, green, and rapid synthesis of silver nanoparticles (Ag-NPs) prepared by biological (green) techniques using *Sargassum muticum* (*S. muticum*). These green-synthesized nanoparticles were examined by ultraviolet-visible spectroscopy (UV–Vis), transmission electron microscopy (TEM), powder X-ray diffraction (XRD) and Fourier transform infrared (FTIR) spectroscopy to determine their size and shape.

## 2. Results and Discussion

**Figure 1:**
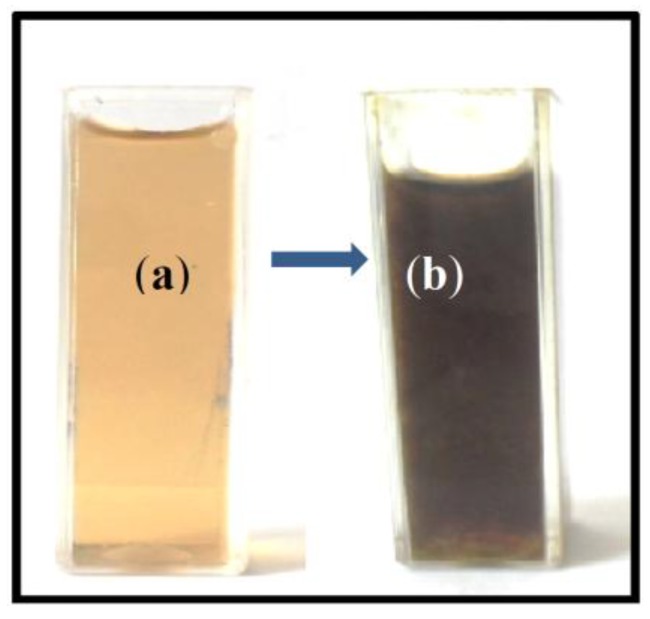
The aqueous extract of *S. muticum* (**a**) before; and (**b**) after synthesis of Ag-NPs.

The formation of silver nanoparticles was confirmed through visual assessment. The reaction mixture turned to dark brown color from brownish-yellow color within 20 min indicated the synthesis of silver nanoparticles (Figure 1). The appearance of dark brown color may be due to the excitation of surface plasmon resonance (SPR) effect and reduction of AgNO_3_ [30].

### Characterization of Ag Nanoparticles

**Figure 2:**
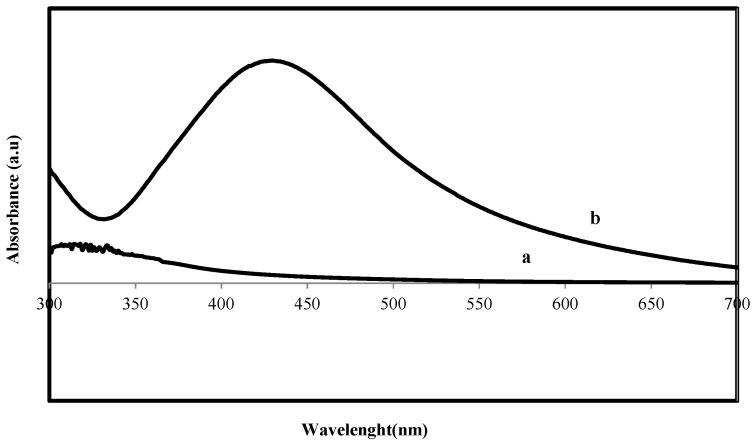
UV–Visible spectrum of (**a**) *S. muticum* aqueous extract; and (**b**) *S. muticum* formed Ag-NPs.

UV–Vis spectrum of reaction mixture at different wavelengths ranging from 300 to 700 nm showed strong absorption peak with centering at 420 nm (Figure 2) indicated the formation of Ag-NPs. This absorption is close to that seen for silver nanoparticles formed by different methods [31]. The wide absorption peak may be induced by the wide size distribution of Ag nanoparticles.

The FT-IR spectra were used to identify the possible biomolecules responsible for the reduction of the Ag^+^ ions and capping of the *S. muticum* formed Ag-NPs.

**Figure 3:**
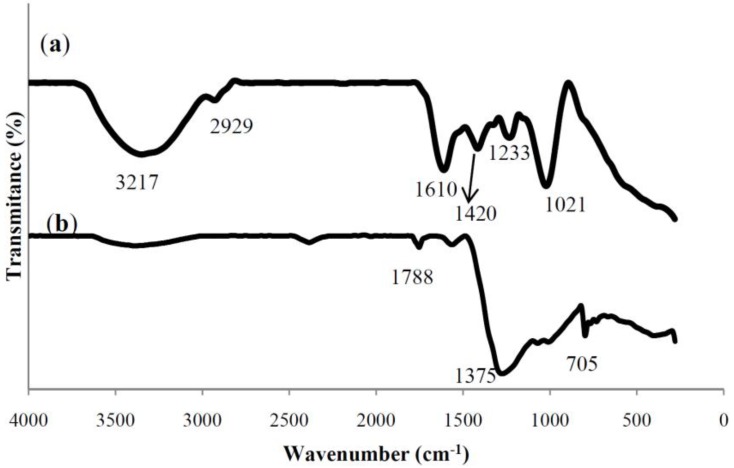
FT-IR spectrum for (**a**) the *S. muticum* a queous extract; and (**b**) *S. muticum* formed Ag-NPs.

Figure 3 shows the FTIR spectra of *S. muticum* aqueous extract and bio-synthesized Ag-NPs.

In the FTIR spectrum of *S. muticum*, the signal at 1235 cm^−1^ corresponded to asymmetric stretching vibration of a sulphate and band at 1021 cm^−1^ attributed to symmetric C–O vibration associated with a C–O–SO_3_ [32] or the C–OH, which disappeared after synthesis of Ag-NPs. This specified the involvement of sulfate or hydroxyl groups in the reduction process of Ag-NPs. In addition, the peaks at 3217 cm^−1^ (OH stretching) and 2929 cm^−1^ (CH stretching) were also detected. After reduction of AgNO_3_, the decreases in intensity at 3217 cm^−1^ imply the involvement of the OH groups in the reduction process. This is further confirmed with a reduction in PH of solution during the reaction. The above peaks generally occur in polysaccharides indicating the participation of sulfated polysaccharides in the synthesis of Ag NPs. This is in agreement with our previous studies, which showed that the sulfated polysaccharides present in *S. muticum* had strong ability to synthesis of Fe_3_O_4_ [19] and ZnO [33] nanoparticles. Similarly, Venkatpurwar and Pokharkar’s (2011) [34] reported that sulfated polysaccharides isolated from the marine alga Porphyra vietnamensis (Rhodophyta) had a strong capacity to synthesize Ag NPs.

The peaks at 1415 cm^−1^ indicate the C–C groups derived from aromatic rings that are present in the *S. muticum* aqueous extract. Another peak at 1610 cm^−1^ is attributed to the stretching vibration of (NH) C=O group that is characteristic of proteins shifted from 1610 cm^−1^ and became shorter after synthesis of Ag-NPs, indicating a member of (NH)C=O group within the cage of cyclic peptides is involved in synthesizing and capping the nanoparticles. In the FTIR spectrum of *S. muticum*, Ag-NPs were formed. The additional peaks at 2466, 1788, 1375, 705, and 391 cm^−1^ are related to Ag-NPs. We propose the presence of vander waals forces of interaction between nitrogen and oxygen atoms in bio-compounds present in *S. muticum* and Ag-NPs. Therefore, the FT-IR results imply that the Ag-NPs were successfully synthesized and capped with bio-compounds present in the *S. muticum* extract by using a green method.

**Figure 4:**
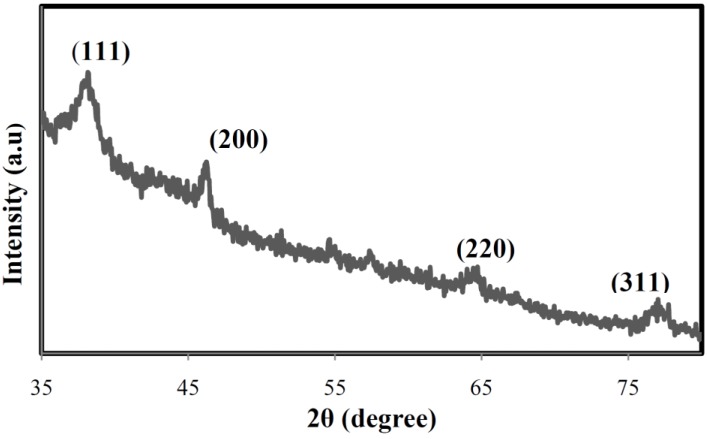
X-ray diffraction pattern of bio-synthesized Ag-NPs.

The XRD pattern (Figure 4) shows that the particles are crystalline with small size. The lattice planes (111), (200), (220), and (311) were identified with the corresponding Bragg’s angles of 37.95°, 45.84°, 64.07°, and 76.43°, respectively, which confirm the face-centered cubic structure of the formed Ag-NPs. The size of formed silver nanoparticles was estimated by using Scherrer’s equation [35] by determining the width of the (111) Bragg reflection and was calculated to be around 8 nm.

**Figure 5:**
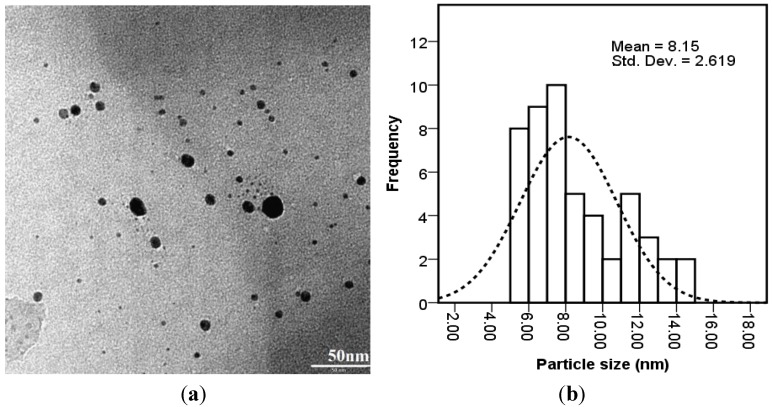
The (**a**) TEM micrograph; and (**b**) particle size distributions of bio-synthesized Ag-NPs.

The TEM image Figure 5a and particle size distribution graph Figure 5b show the Ag-NPs formed were well dispersed with a spherical structures and particle size ranging from 5 to 15 nm with some deviations. Control of the size and structure of the resultant nanoparticles can be related to the interactions between bio-compounds such as polysaccharides, proteins, polyphenols and phenolic compounds and metal atoms [36].

## 3. Experimental Section

### 3.1. Materials

Specimens of the brown marine algae *S. muticum* from the coastal areas of Persian Gulf waters were collected and washed carefully with distilled water. The clean marine alga was freeze dried at −20 °C and then, crushed into powder.

AgNO_3_ (99.98%) was used as silver precursor and it was supplied from Merck (Darmstadt, Germany). All the solutions were prepared with deionized water.

### 3.2. Preparation of *S. muticum* Extracts

Powder marine alga *S. muticum* sample (1 g) was dispersed in 100mL distilled water by magnetic stirring and heated at 100 °C for 20 min. The extract was filtered through mesh, followed by Millipore filter (0.2 μm), and stored at −20 °C before use.

### 3.3. Biosynthesis of Ag-NPs

The Erlenmeyer flask containing 50mL of aqueous solution (1 mM) of AgNO_3_ was reacted with 50 mL of the aqueous extract of *S. muticum* for 30min under continuous stirring at 35 °C and then allowed to stand at room temperature for another 2 h. Initial PH of solution was about 7.5 which changed to 5.6 at the end of the reaction. The dark brown solid product was collected through centrifugation at 6000 rpm for 10 min and careful washing with distilled water. The final products were obtained by drying at room temperature. The resulting dried sample was crushed into powder and stored in an air tight container for further analysis.

### 3.4. Characterization of Ag-NPs

Phase purity and particle size were determined by X-ray diffraction (XRD) analysis recorded by diffractometer (XPERT-PRO) with nickel-filtered Cu (λ = 1.542 Å) at room temperature and the instrument was operated at 40 kv and 30 mA. The powder sample with smooth surface was mounted on sample holder, and the measurement was carried out at 10°–70°. The chemical structure was examined by using FTIR spectrometer (Perkin-Elmer 1725X, Waltham, MA, USA). FTIR spectra of the solution samples were studied with a resolution of 4.0 cm^−1^ at room temperature using a KBr disc containing 1.0 mg of the sample and 0.1 mg of the fine grade KBr at wavenumber range from 400 to 4000 cm^–1^. The shape, size and microstructures of the sample were characterized using a HITACHI H-700 transmission electron microscope (Hitachi, Ltd., Tokyo, Japan) with an acceleration voltage of 120 kV at room temperature. TEM samples were prepared by dispersing small quantities of the dried sample into distillated water and depositing a few drops of the resulting suspension on a copper grid. Size distribution and the average size of 80 nanoparticles were estimated on the basis of three TEM images with the assistance of Sigma-Scan Pro software (SPSS IBM, Statistics 20, IBM Corporation, Endicott, NY, USA). The sample solutions were analyzed at room temperature for UV–Visible absorption using UV–Vis spectrophotometer (a Lambda 25-Perkin Elmer, Waltham, MA, USA). The absorbance spectra were scanned in the range of 200–800 nm with a 1 nm interval at room temperature.

## 4. Conclusions

In this study, a simple, ecofriendly and economic biological procedure has been developed to synthesize Ag-NPs. The Ag-NPs were synthesized by bio-reduction of silver ions using the brown marine algae *S. muticum* aqueous extract. The biosynthesized silver nanoparticles have spherical shapes and the particle size ranges from 5 to 15 nm with a mean size of 8 nm. The FTIR spectra revealed the involvement of sulfate and hydroxyl moieties of polysaccharide in the formation of Ag-NPs. The biosynthesized silver nanoparticles are expected to have remarkable applications in pharmaceutical and biomedical fields.
